# Bioefficacy of ecbolin A and ecbolin B isolated from *Ecbolium viride* (Forsk.) Alston on dengue vector *Aedes aegypti* L. (Diptera: Culicidae)

**DOI:** 10.1016/j.parepi.2016.03.004

**Published:** 2016-04-06

**Authors:** Appadurai Daniel Reegan, Munusamy Rajiv Gandhi, Govindan Sivaraman, Kalaimaran Francina Cecilia, Ramalingam Ravindhran, Kedike Balakrishna, Michael Gabriel Paulraj, Savarimuthu Ignacimuthu

**Affiliations:** a Division of vector control, Entomology Research Institute, Loyola College, Chennai 600 034, Tamil Nadu, India; b National Vector Borne Disease Control Programme, ROH&FW, Govt. of India, Besant Nagar, Chennai 600 090, Tamil Nadu, India; c Department of Plant Biology and Biotechnology, Loyola College, Chennai 600 034, India; d Visiting Professor Programme, Deanship of Research, King Saud University, Riyadh 11451, Saudi Arabia

**Keywords:** Ecbolin A, Ecbolin B, *Ecbolium viride*, Bioassay, *Aedes aegypti*

## Abstract

Ecbolin A and ecbolin B were isolated from ethyl acetate extract of *Ecbolium viride* (Forsk.) Alston root and evaluated for larvicidal and growth disturbance activities against *Aedes aegypti* L. (Diptera: Culicidae). For larvicidal activity, the third instar larvae of *A. aegypti* were exposed to different concentrations viz., 1.0, 2.5, 5.0 and 10 ppm for each compound. Among the two compounds screened, ecbolin B recorded highest larvicidal activity with LC_50_ and LC_90_ values of 0.70 and 1.42 ppm, respectively. In control, the larval behaviour was normal. The active compound ecbolin B was tested for growth disruption activity at sub lethal concentrations viz., 0.5, 1.0 ppm and observed for malformation like larval gut elongation, larval longevity, intermediates, malformed adults, failed adult emergence and compared with methoprene. The results showed significant level of larva–pupa intermediates, pupa–adult intermediates, malformed adult emergence and less adult formation against *A. aegypti*. The histopathological results revealed a severe damage on the midgut epithelial columnar cells (CC) and cuboidal cells (CU) in ecbolin B treated larvae of *A. aegypti*. Similarly peritrophic membrane (pM) was also observed to be damaged in the treated larvae. The present results suggest that, ecbolin B could be used as a larvicidal agent against dengue vector *A. aegypti*.

## 1 Introduction

Mosquitoes are arthropod vectors responsible for transmitting various pathogens and mosquitoes are called as ‘Public Enemy Number One’ (WHO, 1996a). *Aedes aegypti* L. is the primary vector, which is involved in the transmission of arboviruses responsible for major diseases like dengue, dengue hemorrhagic fever, chikungunya and zika (Harrington et al., 2005, Kannathasan et al., 2011). Dengue fever is endemic to many tropical countries including India (Ahmed and Akram, 2005, Valenca et al., 2013).

Mosquito borne diseases are major threat to human health. Currently, biological and chemical compounds like *Bacillus thuringiensis israelensis* (Bti), *Bacillus sphaericus* (BS), pyrethroids, pyriproxyfen, permethrin, diflubenzuran and methoprene are used as larvicidal and growth regulating products in integrated vector management (IVM) against *A. aegypti* (Bellini et al., 2014). Botanical compounds are also a good choice for IVM based control of mosquito larvae (Sutthanont et al., 2010, Madhu et al., 2010, Bayen, 2012, Muthu et al., 2012, Costa et al., 2012). Literature reveals many reports on phytocompounds against vector mosquitoes (Pelah et al., 2002, Jang et al., 2005, Chapagain et al., 2008, Perumalsamy et al., 2009, Han et al., 2013, da Silva Gois et al., 2013). Many authors proved that, phytocompounds primarily affect the biosynthesis or the mechanisms of ecdysone, showed disturbing effects on mosquito larval growth and moulting (Reegan et al., 2014, Sakthivadivel and Thilagavathy, 2003, da Silva et al., 2013, Corzo et al., 2012).

*Ecbolium viride* (Forsk.) Alston (Acanthaceae) is a perennial woody under shrub (also known as Green Shrimp) found in the plains of India and also in Arabia, Malaysia, Sri Lanka and Tropical Africa (Rastogi, 1979, Cecilia et al., 2014). This plant is widely used in Indian traditional medicinal system such as Siddha, Ayurveda, Unani and Folk (Nair et al., 1985, Khare, 2007). In folk medicine, aqueous extract of dried roots of the plant is used for menorrhagia (Datta and Maiti, 1968, Kirtikar and Basu, 1987). The roots of *E. viride* used for the treatment of jaundice (Nair et al., 2007) and rheumatism (Shanmugam et al., 2009); while the roots and leaves together are used against tumour (Yusuf et al., 2009). Further, the extracts obtained from the root of *E. viride* showed a number of pharmacological activities viz., antioxidant (Babu et al., 2011), anti-inflammatory (Lalitha and Sethuraman, 2010), anti-hepatotoxicity (Priyadharshni et al., 2011, Pandey, 2011), antiplasmodial, antitrypanosomal and antimalarial activity (Abdel-Sattar et al., 2009).

In our earlier study, we have reported the larvicidal and pupicidal activities of ecbolin A and ecbolin B isolated from the ethyl acetate extract of *E. viride* root against *Culex quinquefasciatus* (Cecilia et al., 2014). In the present study, the isolated compounds, ecbolin A and ecbolin B were assessed for their effects on *A. aegypti*.

## 2 Materials and methods

### 2.1 Plant collection, extraction and isolation of ecbolin A and ecbolin B

**Fig. 1:**
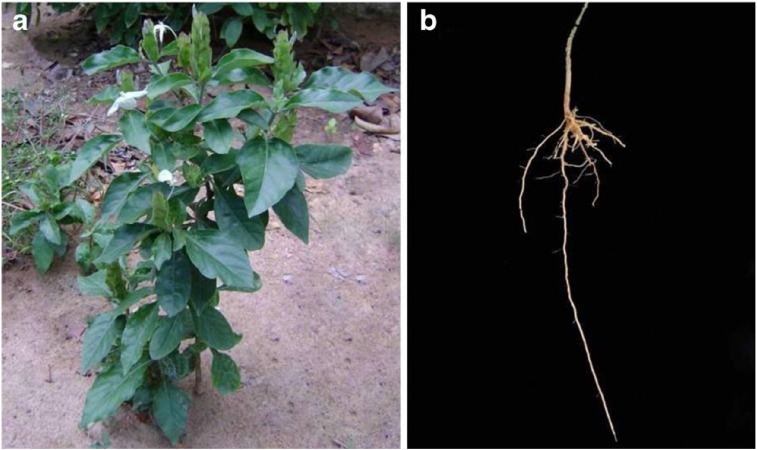
*Ecbolium viride* whole plant (a) and its root (b). Cecilia et al. (2014).

**Fig. 2:**
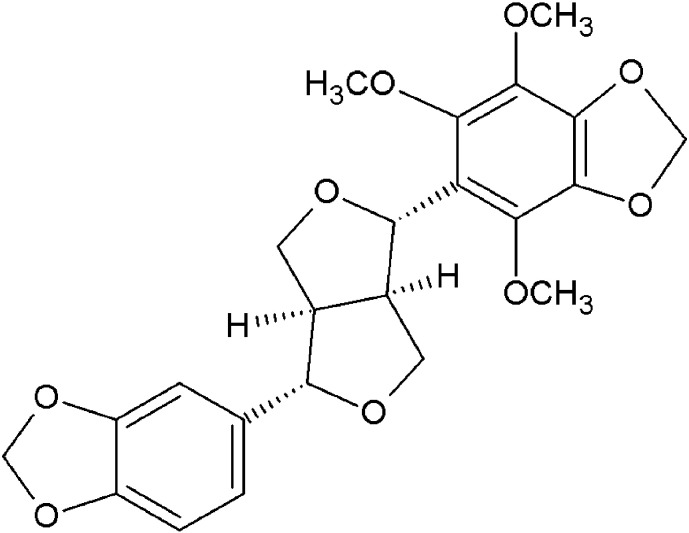
Chemical structure of ecbolin A.

**Fig. 3:**
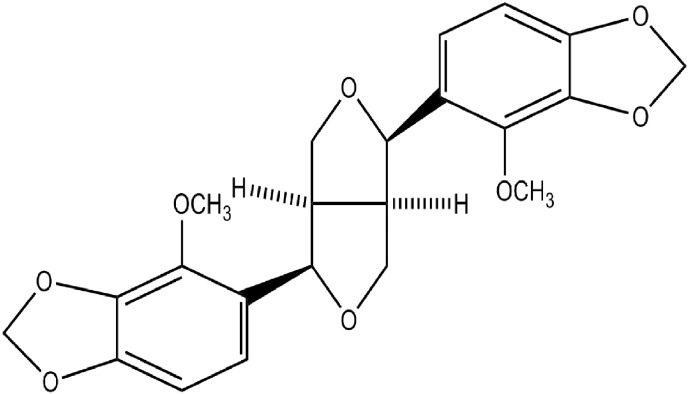
Chemical structure of ecbolin B.

Roots of *E. viride* (Fig. 1) were collected from Srirangam, Trichy, Tamil Nadu, India. The crude ethyl acetate extract (40 g) of *E. viride* root was subjected to column chromatography over silica gel (200 g- Qualigens 100–200 mesh) and eluted with n-hexane followed by combinations of n-hexane: ethyl acetate (95:5 to 0:100) and ethyl acetate: methanol (95:5 to 0:100). The eluted fractions were combined based on the TLC results and finally a total of twelve fractions were obtained. Based on the activity results, the fraction 6 and fraction 7 were selected for further crystallization and identified as ecbolin B and ecbolin A, respectively. A detailed procedure on processing plant material, isolation of ecbolin A and ecbolin B (Fig. 2, Fig. 3) and structural elucidation have been described in our earlier reports (Cecilia et al., 2012a, Cecilia et al., 2012b, Cecilia et al., 2014).

### 2.2 Insect rearing

Larvae of *A. aegypti* were obtained from the stock culture maintained at the Entomology Research Institute laboratory, which were free of exposure to pathogens, insecticides or repellents. Rearing conditions were 27 ± 2 °C temperature, 75–85% relative humidity and a photoperiod of 14 ± 0.5 h.

### 2.3 Larvicidal assay

Larval toxicity assay was performed as per the method prescribed by WHO (2005) with slight modifications. Test concentrations were 1.0, 2.5, 5.0 and 10 ppm for each compound prepared using acetone (1 ml) and five replicates were maintained for each concentration. Groups of twenty third instar larvae (In WHO larvicidal assay: 25 third instar larvae used) of *A. aegypti* were used for each replication. The analytical standard of temephos (98% purity) were purchased from Sigma Aldrich and used in the same concentrations with five replicates as positive control. Five controls were maintained, consisting of 1 ml acetone in 249 ml water. Same amount of acetone (1 ml), which was used to dissolve compound was added in control to ensure that the larval mortality in treatments is due to the compounds. The dead larvae were registered after 24 h exposure period. The percent mortality was calculated for each concentration using the formula (A.1) and corrections for mortality were done using formula (A.2) of Abbott's (Abbott, 1987).

(A.1) Percentage of mortality:

$$\frac{\mathrm{No}.\text{of Dead larvae}}{\mathrm{No}.\text{of Larvae introduced}}\times 100$$

(A.2) Corrected percentage of mortality:

$$1-\frac{n\;\text{in}\;T\;\text{after treatment}}{n\;\text{in}\;C\;\text{after treatment}}\times 100$$

Where *n* is the number of larvae, T is the treated and C is the control.

### 2.4 Growth disruption assay

Based on the larvicidal activity results, the effective compound ecbolin B was used to study the growth disruption (morphological malformation) factors such as larvae–pupae intermediate, larval gut elongation, larval longevity, pupae–adult intermediate, malformed adults and failed adult emergence at sub lethal concentrations viz., 0.5 and 1.0 ppm following the method of Reegan et al. (2014). Five replicates of treated and control were maintained with twenty third instar larvae in each. Methoprene was used as positive control. Abnormalities were observed 24 h post-exposure of the larvae till adult emergence. The larvae were fed with yeast and dog biscuits (4:6 ratio).

### 2.5 Histopathological effects

The *A. aegypti* larvae, treated with ecbolin B at 10 ppm concentration were subjected to histopathological study. For this, the treated and control larvae were fixed in Carnoy 2 for 72 h as per the method of Raymond et al. (2007). The dehydration of tissue was performed sequentially with alcohol viz., 50, 60, 70, 80, 90 and 100% for every 2 h. Then the samples were placed in xylene for 6 h and transferred to warm oven with wax for embedding for about 2 h. The liquid wax was poured in paper boats with sample, cooled and wax blocks were prepared. Then sectioning was made with the microtome (Minot microtom models “Stiasnie”) at 8 μm.

Then the sections were placed on clean slides to adhere and left undisturbed for 24 h. Further, de-waxing was done with xylene for 5 min and the hydration of sectioned tissue was performed sequentially with alcohol viz., 100, 90, 80, 70, 60, 50% and then with distilled water. Then it was stained in Ehrlich's haematoxylin and again dehydration was made sequentially with alcohol viz., 50, 60, 70, 80, 90 and 100% and counterstained with eosin. A single wash was made in alcohol (100%) and two dips were made in xylene and then mounted with one drop DPX. The observation was made with the microscope (Motic images plus 2.0 ML) connected to a computer and midgut cells of the treated and untreated larvae of *A. aegypti* were photographed. Observations were made on epithelial columnar cells (CC), cuboidal cells (CU), peritrophic membrane (pM), nucleus (N), midgut content (MC) and ectoperitrophic space (ES), muscles (M) of treated larvae for any damage caused by ecbolin B and compared with control.

### 2.6 Statistical analysis

The corrected percentage (using Abbott's formula) mortality values for each concentration of larvicidal data were subjected to probit analysis (US EPA probit analysis software; version 1.5) to estimate lethal concentrations (LC_50_ and LC_90_ values) and the differences were considered as significant at P ≤ 0.05. The confidence interval (95%) for LC_50_ and LC_90_ values was also produced from the corrected percentage values by Abbott's formula. Kaplan–Meier comparison of survival with control was compared using SPSS program (Version: 20.0) to support probit analysis. The calculated growth disruption rate were analysed in Graph Pad Prism version 5.0 for Windows, Graph Pad Software, San Diego, CA, U.S.A.

## 3 Results

### 3.1 Larvicidal activity of ecbolin A and ecbolin B

The results of larvicidal activity of ecbolin A and B against *A. aegypti* larvae are given in Table 1. Concentration-dependent mortality was observed. The highest concentrations (5 ppm and 10 ppm) of ecbolin B showed 100% larvicidal activity against *A. aegypti* in 24 h. The LC_50_ and LC_90_ values of ecbolin B were 0.70, 1.42 ppm against the third instar larvae of *A. aegypti*, respectively (Table 1).

**Table 1:** Lethal concentrations (in ppm) of ecbolin A and B against *A. aegypti* larvae compared with azadirachtin and temephos.

Mosquito species	Treatment	Mortality in control	LC_50_ (ppm)	95% confidence limit		LC_90_ (ppm)	95% confidence limit		Slope ± SE	Intercept ± SE	χ^2^
		(Total)		LL	UL		LL	UL			
*Aedes aegypti*	Ecbolin A⁎	1 (100)	8.56	7.67	9.44	17.47	15.35	20.79	4.1 ± 0.4	1.1 ± 0.4	2.9#
	Ecbolin B⁎⁎⁎		0.70	0.63	0.77	1.42	1.28	1.62	4.2 ± 0.3	5.6 ± 0.1	4.8#
	Temephos⁎⁎		1.10	0.21	1.89	2.31	1.38	3.18	3.9 ± 0.4	5.1 ± 0.1	5.8#

LC_50_-lethal concentration that kills 50% of the exposed larvae; LC_90_-lethal concentration that kills 90% of the exposed larvae; LL-lower limit (95% confidence limit); UL-upper limit (95% confidence limit). Control value was included in the Abbott's formula for correction. Kaplan Meier comparison of survival with control.

# p ≤ 0.05, level of significance of chi-square values.

⁎ p ≤ 0.05.

⁎⁎ p ≤ 0.01.

⁎⁎⁎ p ≤ 0.001.

*A. aegypti* larvae were less susceptible to ecbolin A in the present assessment and the LC_50_ and LC_90_ values of ecbolin A on larval mortality were 8.56, 17.47 ppm against the third instar larvae of *A. aegypti*, respectively (Table 1). The LC_50_ and LC_90_ values of temephos on mortality were 1.10, 2.31 ppm against the larvae of *A. aegypti*, respectively. Restless movement and convulsion were observed in all the exposed concentrations of ecbolin B and dead larvae were settled down as reported earlier (Reegan et al., 2013), whereas in control (1 ml acetone in 249 ml water) the larval behaviour was normal. Chi-square values were significant at ^⁎^p ≤ 0.05.

### 3.2 Growth disruption activity of ecbolin B

**Fig. 4:**
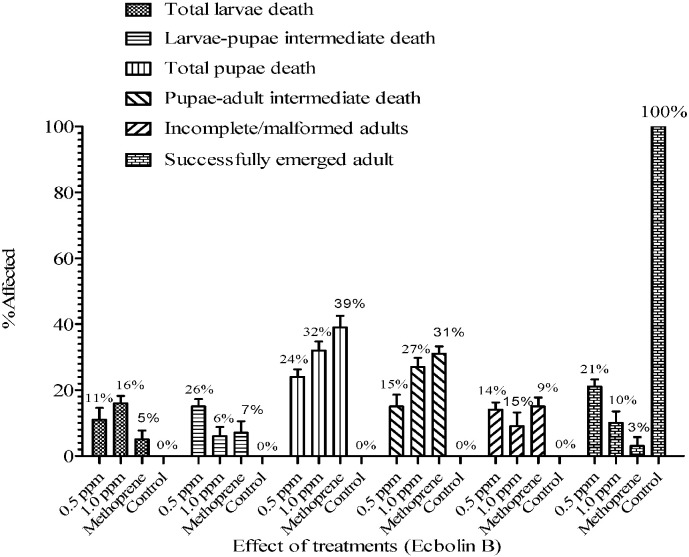
Proportion of larval deformity and mortality in *A. aegypti* upon exposure to ecbolin B (0.5 and 1.0 ppm) compared with methoprene (1.0 ppm) and control.

In the present study, larva–pupa intermediates, pupa–adult intermediates, incomplete/malformed adult emergence were observed and less adult formation was observed with ecbolin B at sub lethal concentrations (Fig. 4). The positive control methoprene also recorded malformation occurred mainly at the pupal stage and adult emergence was strongly inhibited at 1.0 ppm concentration (Fig. 4). But in control, the development was normal and 100% adult emergence was observed (Fig. 4). Gut elongation and larval longevity were not observed during the present investigations. These observations suggest that the ecbolin B contributing significant level of growth disturbance and deformities at sub lethal concentrations against *A. aegypti*.

### 3.3 Histopathological effects of ecbolin B on larval midgut cells

**Fig. 5:**
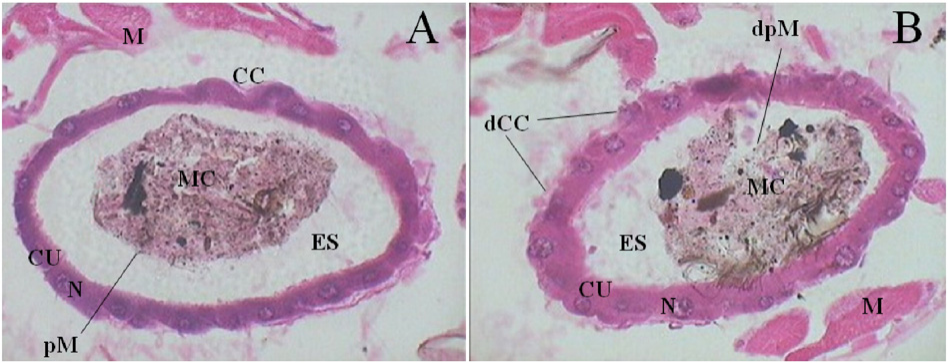
Cross section parts of midgut of 3rd instar larvae of *A. aegypti* treated with ecbolin B (B) compared with control (A). Columnar cells (CC), damaged columnar cells (dCC), cuboidal cells (CU), nucleus (N), peritrophic membrane (pM), damaged peritrophic membrane (dpM), midgut content (MC), ectoperitrophic space (ES), muscle (M).

The histopathological results revealed a severe damage on the midgut epithelial columnar cells (dCC) of treated larvae, which was exposed to ecbolin B at 10 ppm concentration (Fig. 5B). Similarly peritrophic membrane (dpM) was also observed to be ruptured in treated larvae of *A. aegypti* and the midgut content (MC) was oozed out into the ectoperitrophic space (ES); but in control, peritrophic membrane (pM) was normal in appearance and midgut content (MC) was observed to be tightly packed inside peritrophic membrane (Fig. 5A).

## 4 Discussion

Plant extracts and isolated compounds would be a valuable alternative to synthetic chemical insecticides and could be used in integrated vector management programmes (Kabir et al., 2013).

Ecbolin is a lignan compound and in the present study ecbolin B was effective and showed strong larvicidal activity than ecbolin A against *A. aegypti* (Table 1). Similarly, Park et al. (2005) identified two lignan constituents from *Phryma leptostachya* Var. *asiatica* roots namely leptostachyol acetate and 8′-acetoxy-2,2′,6-trimethoxy-3,4,4′,5′-dimethylenedioxyphenyl-7,7′-dioxabicyclo [3.3.0]octane. Their study revealed that leptostachyol acetate was effective and showed LC_50_ value of 0.41, 2.1, and 2.3 ppm against *Culex pipiens pallens*, *A. aegypti*, and *O. togoi*, respectively. In another study, three lignans namely, phrymarolin-I, haedoxane A, and haedoxane E were isolated from the petroleum ether extract of *Phyrma leptostachya* L and tested for larvicidal activity against *C. pipiens pallens*. The toxicity of each compound varied with LC_50_ value of 1.21, 0.025, and 0.15 ppm for Phrymarolin-I, haedoxane A, and haedoxane E, respectively against the early fourth instar larvae of *C. pipiens pallens* (Xiao et al., 2012). In another study, a lignan was identified as grandisin from *Piper solmsianum*, which showed 80% and 100% larval mortality against *A. aegypti* at 10 mg/ml and 100 mg/ml, respectively (Cabral et al., 2009). In a study, Batallán et al. (2013) reported the larvicidal activity of a lignan nordihydroguaiaretic acid against *C. quinquefasciatus* with LC_50_ value of 0.092 mg/ml. Kishore et al. (2011) reviewed the efficacy of plant derived lignans against mosquito larvae. Further, the larvicidal activity of ecbolin B (LC_50_–0.70 ppm) was comparable to temephos, which showed LC_50_ value of 1.10 ppm during the present study (Table 1).

In the present study, deformities like larva–pupa intermediate, pupa–adult intermediate and incomplete adult emergence were recorded (Fig 4). Similar to our result, Nathan et al. (2008) has demonstrated the growth and moulting disrupting effects of two triterpenoids viz., 3β,24,25-trihydroxycycloartane and beddomeilactone isolated from the leaves of *Dysoxylum malabaricum* and *Dysoxylum beddomei* against *Anopheles stephensi* at 1.0 and 2.5 ppm concentrations. In another study, incomplete adult emergence was reported with *Copaifera* sp. oil at 48 mg/l concentration against *A. aegypti* (Prophiro et al., 2012). Similarly, Nayar et al. (2002) evaluated the growth regulatory activity of s-methoprene and the results varied among different mosquito species. In their study, the maximum emerging inhibition of 84% and 44.3% recorded at 0.4 and 0.05 ppm with *C. quinquefasciatus* and *Aedes albopictus*, respectively. da Silva and Mendes (2007) reported abnormalities like dead pupae, larva-like abdomen, pupal–adult intermediate with methoprene at 70 ppb concentration against *A. aegypti*.

## 5 Conclusion

In summary, the isolated compound ecbolin B from ethyl acetate extract of *E. viride* root showed the highest larval mortality at the lowest concentration than ecbolin A against *A. aegypti* larvae. Ecbolin B also exhibited intermediates and malformed adults at sub lethal concentrations. The histopathological results revealed a severe damage on the midgut epithelial columnar cells (CC) and cuboidal cells (CU) of ecbolin B treated larvae. The compound ecbolin B could be used as an effective larvicidal agent for the control of *A. aegypti* mosquitoes. The field application of Ecbolin B through integrated vector management programme would be target specific and environmental friendly.

## Conflict of interest

Authors do not have any conflict of interest.
